# Treatment of Acne Scar Contraction Using Autologous Cultured Epithelial Sheet Transplantation: A Report of Four Cases

**DOI:** 10.7759/cureus.87296

**Published:** 2025-07-04

**Authors:** Tomoka Hyoudou, Yoshie Hirose, Chiharu Fujita, Hajime Inoue

**Affiliations:** 1 Dermatology, Ginza Yoshie Clinic, Tokyo, JPN; 2 Regenerative Medicine, Laboratory of Cell Applied Technologies Co., Tokyo, JPN; 3 Plastic Surgery, St. Marianna University School of Medicine, Kawasaki, JPN

**Keywords:** acne vulgaris therapy, autologous cultured epithelium, epithelial sheet grafting, regenerative medicine therapies, scar contracture, scar treatment

## Abstract

Acne vulgaris often results in disfigurement with scarring if early treatment is insufficient. In particular, tissue destruction due to infection of hair follicles leads to contracture. There are many treatments for acne scars and scar contracture, but therapy of scar contracture, especially ice pick-like scars or crater-shaped scars, is difficult. In this study, we attempted treatment with grafting of an autologous cultured epithelial sheet in four patients with acne vulgaris accompanied by scar contracture. As a result, not only was the scarring improved, but the skin texture was also improved in all cases three months after grafting. Excessive collagen synthesis during the healing process following tissue destruction induces scar contracture, but various proteases secreted by the grafted cultured epithelium are thought to metabolize this excess collagen, improving the scarring and skin texture. Autologous cultured epithelium grafting, which can be completed with a single treatment, may be an effective method for treating acne scars.

## Introduction

Cultured epithelium grafting is an excellent treatment technique for extensive skin defects such as burns and skin dysmorphism with a large treatment area [1]. In particular, in the case of diseases limited to the superficial dermis such as vitiligo vulgaris, cultured epithelium grafts containing melanocytes enable scarless healing [2]. However, deep dermal defects often leave scars and lead to scar contracture [3]. Scar not only affects the appearance but also causes poor skin properties as well as poor skin flexibility [4]. Direct cultured epithelium grafting to severe burn wounds that reach the deep dermis, such as deep dermal burn and deep burn, is unlikely to survive. Therefore, reconstruction of the dermis is necessary, and cadaveric skin grafts and artificial dermis are used [5]. However, the survival rate is low, and hypertrophic scars and scar contracture are often involved scar [6]. The proliferation of collagen tissue is essential for the repair of wounds with dermal defects, but excessive proliferation of collagen or disordered arrangement of collagen fibers can cause scars or scar contracture.

Skin injury with scar contracture is not limited to burns and skin defects with trauma or other factors. Acne, which is common during adolescence and young adulthood, often also leaves behind typical scars when it becomes severe, causing great mental distress [7]. The acne associated with hair follicle infection often extends to the deeper dermis, not only delaying wound healing but also causing scarring and cicatricial contracture due to abnormalities in connective tissue production.

Acne, which is common during adolescence and young adulthood, often leaves behind typical scars when it becomes severe, causing great mental distress [7]. Acne is caused by bacterial infections associated with sebum deposition, but poor lifestyle habits and inadequate skin care can lead to repeated recurrences, worsening the condition and leaving scars. Depressed scars and cyst healing on the face are particularly common, but ice pick-shaped craters that reach the deep dermis with abscesses are difficult to treat. Currently, treatments for severely depressed acne scars include chemical peels, fractional laser, and dermapen therapy in the expecting of remodeling the dermis, but both require long-term treatment and there is a limit to how much improvement can be achieved [8].

In the early stages, cultured epithelium has been used in critical care for extensive severe burns, but it has been reported that cultured epithelium grafting can improve scar contractures associated with burns, except in movable areas such as joints [4]. This may contribute to improving the texture and appearance of scars caused by trauma, not just burns. In this study, many patients with severe scars from acne vulgaris suffer from psychological distress due to cosmetic concerns. Here, we report the results of four cases in which cultured epidermal transplantation was attempted to treat acne scars.

## Case presentation

Patients

This study has been reviewed and approved by a third-party organization as described in the Disclosure section. The indications for autologous cultured epithelium were four patients with skin dysmorphism (pigment anomaly (vitiligo, etc.), scarring after trauma, scar contracture, and functional impairment due to ulcers). All four cases met the following inclusion criteria and did not violate the exclusion criteria. The inclusion and exclusion criteria are shown in Table 1.

**Table 1:** Inclusion and exclusion criteria The above standards have been approved by a third-party organization that reviews regenerative medicine.

Inclusion criteria
(1) Patients in whom the skin harvesting required for autologous skin grafting would be extensive and would be highly invasive when treating the disease
(2) Patients in whom cultured epithelium grafting is expected to improve cosmetic and functional outcomes compared to conventional skin grafting
(3) In principle, patients aged 20 years or older. In the case of those under 20 years of age, those who wish to receive regenerative medicine, etc., and those who can obtain consent from their legal representative
Exclusion criteria
(1) Patients with malignant tumors or infections on the wound surface (ulcerated area)
(2) Patients whose general condition was judged by a doctor to be inappropriate for cultured epidermal grafting
(3) Patients whose general condition was judged by a doctor to be inappropriate for cultured epidermal grafting on the application site
(4) Patients who were unable to follow the doctor's instructions
(5) Patients who did not consent to the treatment

Epithelial cell culture

Skin for epithelial cell culture was collected from the inguinal region. Epithelial cells were cultured by using the Boyce and Ham method [9] and then induced to an epithelial sheet using the method of Rheinwald and Green [10] after confluence. The cultured epithelial sheet was stored on a cell sheet grafting carrier (ATTRAN, Nikkan Industrial Co., Tokyo, Japan) until grafting.

Cultured epithelial sheet grafting and post-grafting management

**Figure 1:**
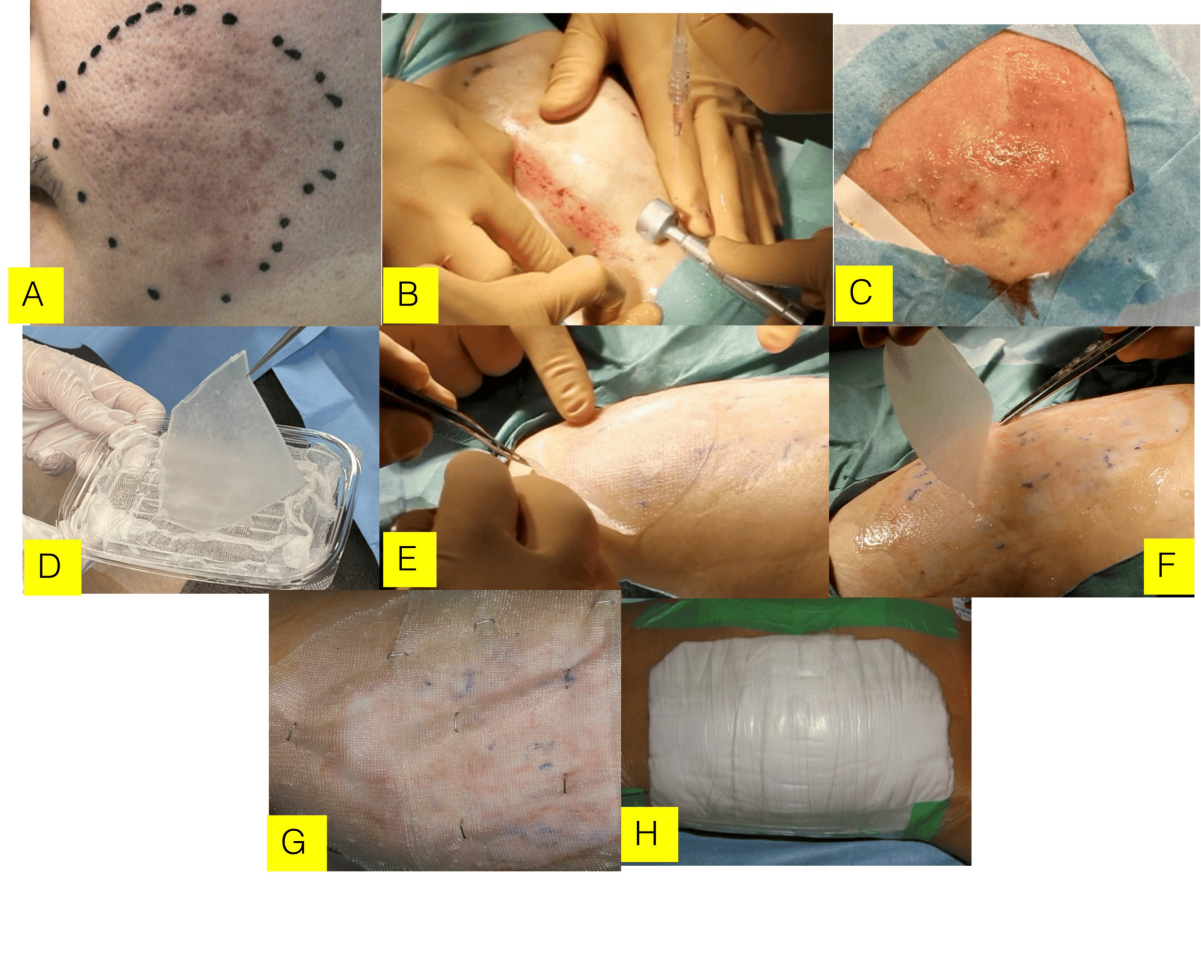
A typical procedure for cultured epithelial sheet grafting for scars and scar contractures The procedure is performed in the order of the alphabet under local anesthesia: A: grafting region, B: abrasions, C: after abrading, D: cultured epithelial sheet, E: grafting, F: peel of the carrier on the epithelial sheet, G: primary dressing, H: secondary dressing. The depth of skin abrasion is limited to spot bleeding (C). The cultured epithelial sheet is peeled with the carrier and transported (D). If necessary, FGF-2, etc., is used to reconstruct the dermis layer simultaneously. The grafted wound is managed using the wet-to-dry technique.

Under local anesthesia, the grafting area was abraded using ground wheels to the extent that pinpoint bleeding was observed in the superficial dermis. An indurated lesion was excised using scissors. Fibroblast growth factor-2 (FGF-2: Fiblast spray, Kissei Co., Tokyo, Japan) was then applied to the abraded wound, and the cultured epithelial sheet was grafted. The grafted wound was covered and protected with non-adherent gauze (Adaptic: Johnson & Johnson Medical Inc., Arlington, TX) coated with white petrolatum. The grafting site was immobilized and rested for one week. The standard procedure is shown in Figure 1.

After confirming that there was no infection or shedding of the grafted epithelial sheet with the first dressing after grafting, showering was permitted. Two weeks after grafting, the grafted surface was actively exposed to the outside air to ensure complete epithelialization and to improve the strength of the grafted skin. If symptoms such as itching occurred at the grafted wound, a steroid ointment was used. Post-therapeutic evaluation was performed using the method of Goodman and Baron [11]. That is, acne symptoms were divided into four kinds of grades (1: Macular, 2: Mild, 3: Moderate, and 4: Severe) on various skin conditions. Secondly, the severity conditions were scored by observation of the same doctor.

Case 1

Case 1 was a 32-year-old man with a chief complaint of acne vulgaris contracture. 

Present Illness

Topical medications have been used for acne treatment under health insurance. No medications were in use at the visit. The patient preferred cultured epithelial sheet grafting over Dermapen treatment, which would require frequent visits.

Symptoms at the First Visit

Depressive-shaped and icepick-shaped scar contracture were found on both cheeks and the tip of the nose.

Postoperative Progress

**Figure 2:**
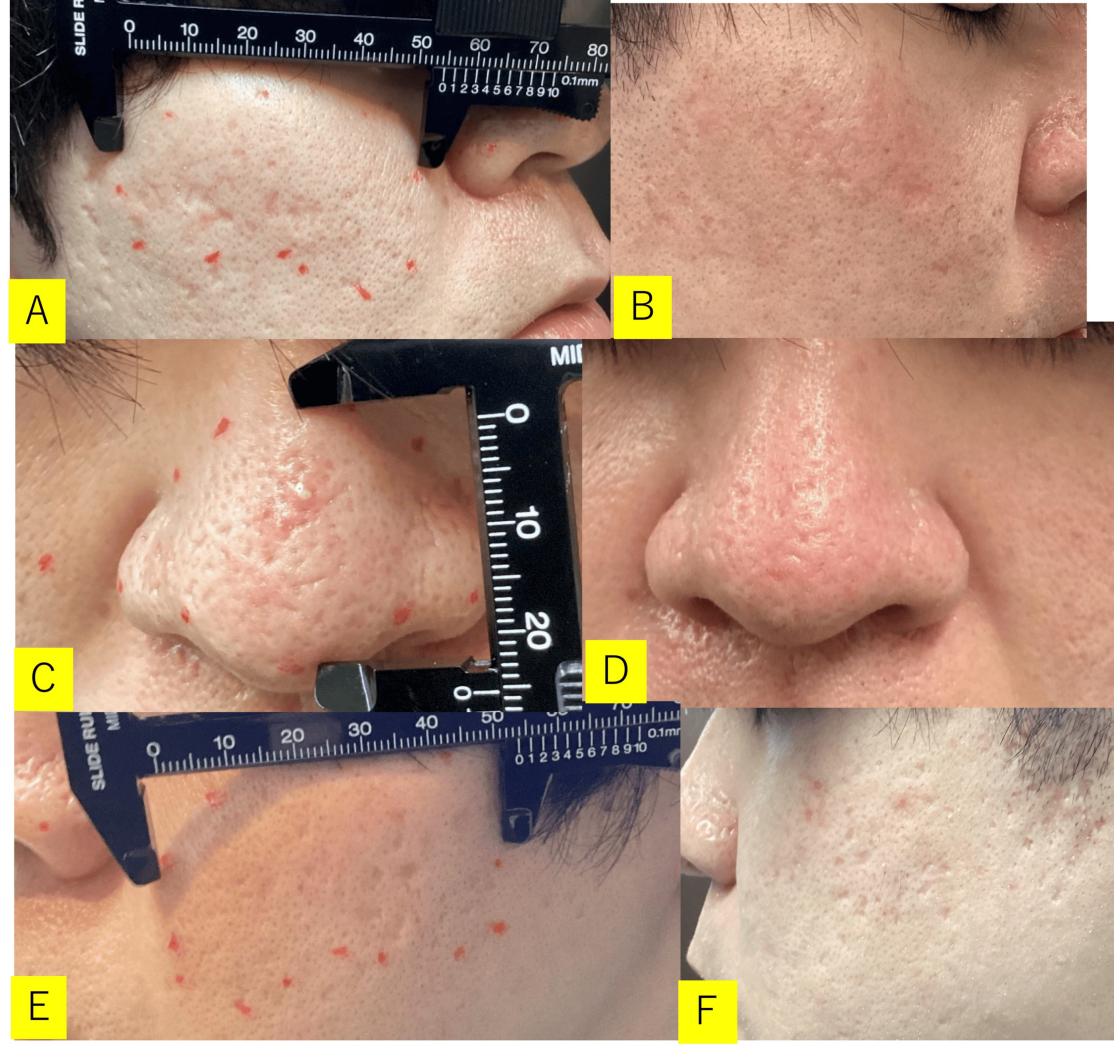
Case 1 (32 y/o, Male) Chief complaint: Acne scar contracture. Depressive-shaped and icepick-shaped scar contracture were found on both cheeks and the tip of the nose. A: before grafting (right cheek), B: two months after grafting (right cheek), C: before grafting (nose), D: two months after grafting (nose), E: before grafting (left cheek), F: two months after grafting (left cheek). Postoperative progress: One month later, the scabs remained, but after three months, they had all peeled off and the crater-shaped scar contracture had improved. After two months, the crater findings on the face had improved continuously

One week after grafting, the cultured epithelial sheet had taken well without infection. The dressing was replaced according to the ordinary method. Two weeks later, some scabs were observed on the grafted wound. One month later, the scabs remained, but after three months, they had all peeled off, and the craters shaped scar contracture had improved. The patient also requested cosmetic treatment of the facial skin. These treatments were planned to be performed after six months. After six months, the crater findings on the face had improved continuously. Treatment of rubor, clogged sebum of hair follicles, and inflammatory acne associated with facial acne was started, but the patient did not visit the hospital regularly for the next six months (one year after surgery). Pre- and postoperative assessments and changes are described in Figure 2.

Case 2

Case 2 was a 32-year-old man with a chief complaint of acne vulgaris contracture. 

Present Illness

Acne was frequent when the patient was about 21 years old. Currently, only a few acnes appear, but these acnes get worse and sometimes require incision. Fractional laser, Dermapen, and Subcision have been performed about 10 times, but the acne scars have not improved. Since Dermapen has been ineffective, the patient requested cultured epithelial sheet grafting. 

Present Condition at the First Visit

Icepick-shaped and depressive-shaped crater acne scar with contracture were found on both cheeks. 

Postoperative Progress

**Figure 3:**
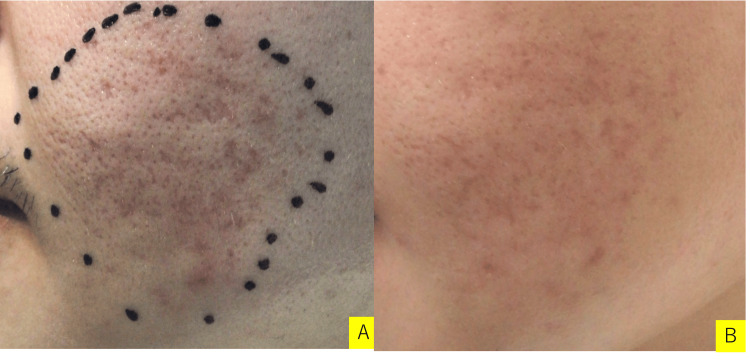
Case 2 (29 y/o, Male) Chief complaints: Acne and acne scar. Icepick-shaped and depressive-shaped crater acne scar with contracture. A: before grafting, B: three months after grafting. Postoperative progress: One month later, the redness became slightly, and the crater-like scar at the grafted site improved. Three months later, the redness improved, and the crater scar had further improved. As a postoperative management, we proposed Subcision in the hope of a synergistic effect.

One week after grafting, the cultured epithelial sheet had taken well, so the hook was removed (Figure 3). Two weeks later, redness was observed on the left cheek, and there was redness at the site of the hook removal. One month later, the redness became slightly, and the crater-like scar at the grafted site improved. Three months later, the redness is improving, and the crater scar has improved. As a postoperative management, we proposed Subcision in the hope of a synergistic effect. Pre- and postoperative assessments and changes are described in Figure 3.

Case 3

Case 3 was a 24-year-old man with a chief complaint of crater-shaped scars.

Present Illness

Various therapies (Dermapen, Potenza, and Fractional laser) on acne scar had been attempted. As there was no effective feeling on these therapies, autologous cultured epithelial sheet grafting was selected.

Present Condition at the First Visit

Crater-shaped acne scars and contracture on both cheeks, mainly box-shaped and ice pick-shaped, were found.

Postoperative Progress

**Figure 4:**
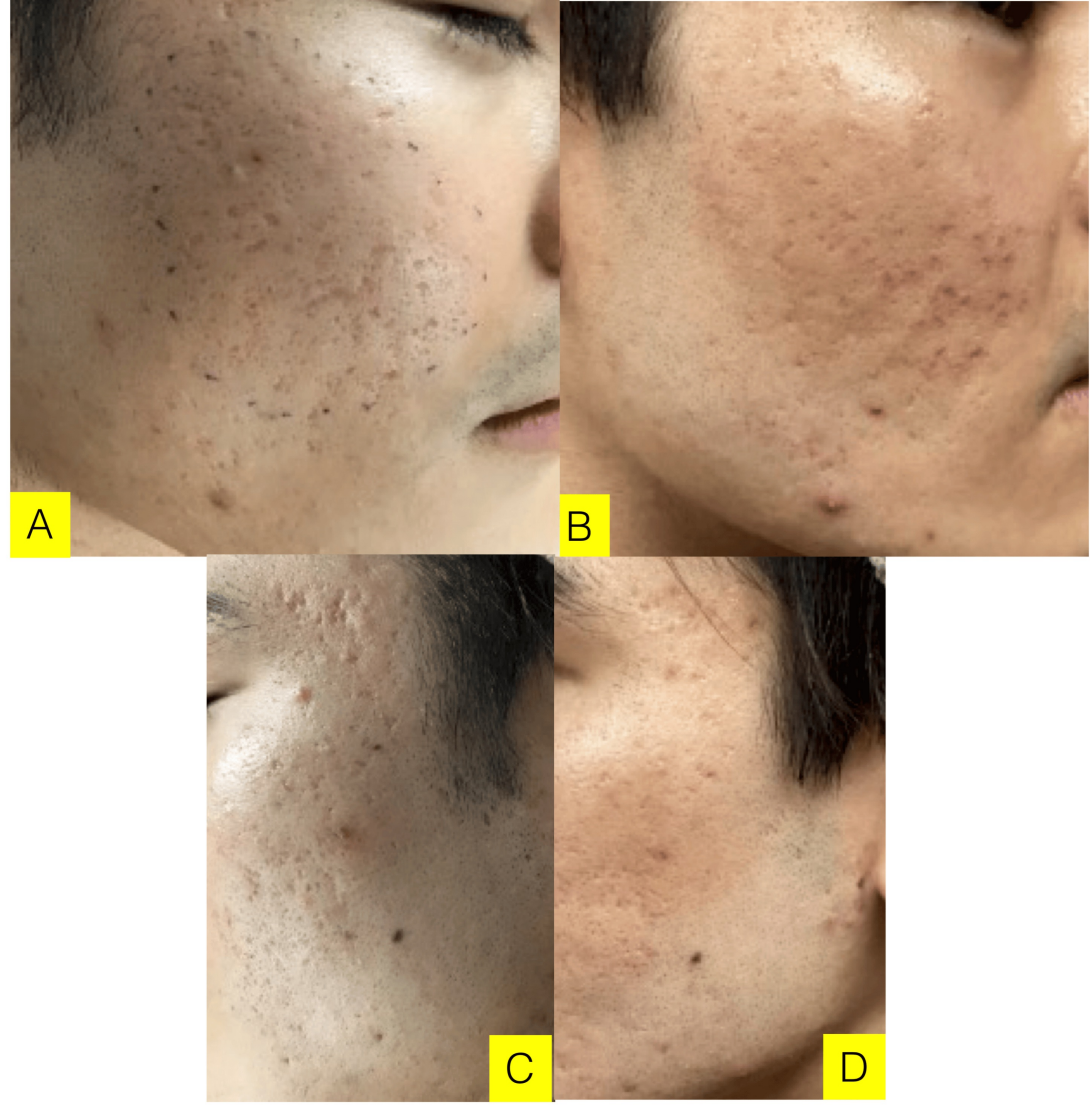
Case 3 (29 y/o, Male) Chief complaint: Crater-shaped scars. Crater-shaped acne scars and contracture on both cheeks, mainly box-shaped and ice pick-shaped, were observed. A: before grafting (right cheek), B: three months after grafting (right cheek), C: before grafting (left cheek), D: six months after grafting (left cheek). Postoperative progress: One month later, redness remained slightly at the grafting site. As a new inflammatory acne was found surrounding the grafting site, an antibacterial topical drug was used. Three months later, the crater-shaped scars had improved and the pores of hair follicle had shrunk, but pigmentation was observed on the ice pick-shaped crater scars due to the TCA cross therapy performed at another hospital. Six months later, not only the crater scars, which are a chief compliant, but also the pigmentation had improved.

One week after grafting, there was redness, but the cultured epithelium had taken well (Figure 4). Two weeks later, redness remained at a few sites after the hook removal. One month later, redness remained slightly at the grafting site. As a new inflammatory acne was found surrounding the grafting site, an antibacterial topical drug was used. Three months later, the crater-shaped scars had improved, and the pores of hair follicles had shrunk, but pigmentation was observed on the ice pick-shaped crater scars due to the TCA cross therapy performed at another hospital. Six months later, not only the crater scars, which are a chief complaint, but also the pigmentation had improved. As the treatment methods to prevent acne were consulted, a combination therapy of laser and peeling techniques was introduced. Pre- and postoperative assessments and changes are described in Figure 4.

Case 4

Case 4 was a 40-year-old woman with a chief complaint of crater-shaped acne scars.

Present Illness

Potenza and Dermapen treatments had been attempted many times, but with little effect. Other therapeutic approach was discussed. A combination of Dermapen and Subcision therapy or autologous cultured epithelial sheet grafting was introduced. Cultured epithelium grafting was requested, as it is expected to be effective with one treatment.

Present Condition at the First Visit

A depression-shaped crater scar was observed on the temple and cheek

Postoperative Progress

**Figure 5:**
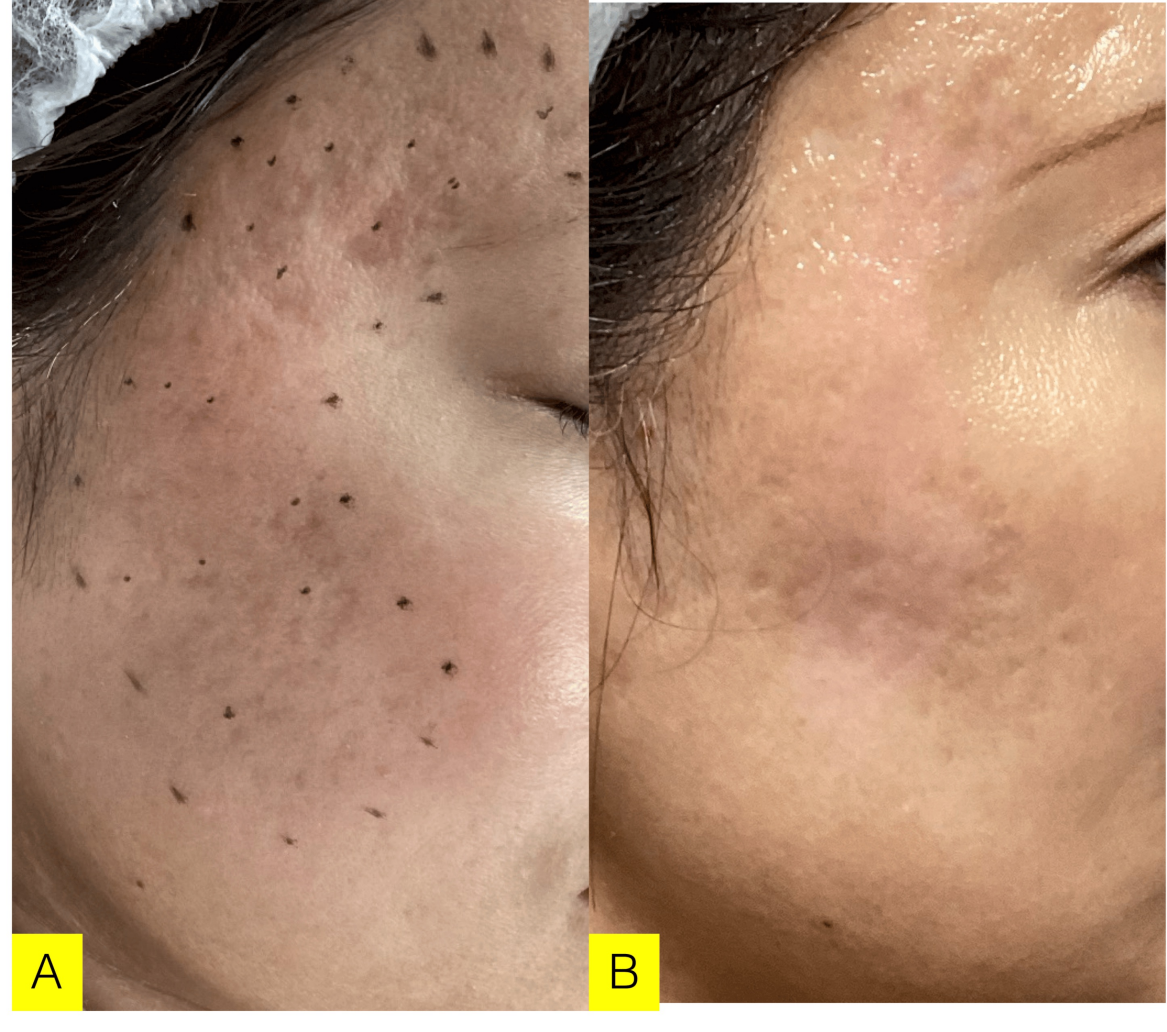
Case 4 (40 y/o, Female) Chief complaint: Crater-shaped acne scar. Depressive-shaped crater scar on temple and cheek. A: before grafting, B: three months after grafting. Postoperative progress: Two weeks later, redness remained, but the acne scar had improved. One month later, the redness at the grafting site was showing signs of further improvement. The crater-shaped acne scar had improved. Three months later, the redness at the grafted site continued to improve.

One week after surgery, there was redness, but the cultured epithelium had taken well. Two weeks later, redness remained, but the acne scar had improved. One month later, the redness at the grafting site was showing signs of further improvement. The crater-shaped acne scar had improved. Three months later, the redness at the grafted site continued to improve. Subcision was proposed due to adhesion between the temple and cheek. Pre- and postoperative assessments and changes are described in Figure 5.

## Discussion

Characteristics of acne scars

Acne is one of the skin diseases that commonly occurs during adolescence. Propionibacterium acnes (P. acnes) is a normal bacterium present on the skin, but it proliferates infectiously within hair follicles, causing inflammation [12]. This is because P. acnes digests triglycerides secreted from sebaceous glands and produces inflammatory free fatty acids. Therefore, suppressing the secretion of sebum leads to the prevention of acne, but in adolescence, the secretion of testosterone increases due to changes in endocrine function, and 5α-reductase (type I) present around the hair follicles converts this testosterone into DHT (dihydrotestosterone), activating the sebaceous glands and increasing sebum secretion [13].

Inflammation of the hair follicle causes the hair follicle to become cystic, ultimately inducing the destruction of the hair follicle wall, but if this inflammation persists and the inflammatory reaction is extremely, it can lead to the destruction of skin tissue and construction of acne scars, causing scar contraction such as crater-shaped and ice pick-shaped on face [14].

Characteristics of scars

When tissue destruction with an inflammatory reaction is severe, secondary healing is required due to the defect of connective tissue in the wound. If excessive production of collagen, which is used to fill the connective tissue, occurs, fibrous healing is induced. This healing mechanism causes scar contractures well as hypertrophic scars, as the collagen filling the wound creates tension that contracts the wound. This type of wound healing often results in loss of functionality and leaves a disfigurement [15].

Split-thickness or full-thickness skin grafts are used to release scar contracture and treat mature scars for cosmetic purposes, but a sufficient area of skin as a donor site is required in a normal site for skin grafting [16].

Characteristics of cultured epithelium

Cultured epithelium is a useful tool for treating extensive skin defects, but the presence of dermis greatly affects the success of grafting. In cases of defects up to the superficial dermis, good survival is expected, and scarring is not noticeable. However, in cases of defects reaching the deep dermis or full-thickness skin, survival of cultured epithelium is not expected [5]. Therefore, cultured epithelium grafting for scar contracture after extensive burns is an effective technique because it reduces the invasiveness of the donor for skin grafting, but it requires reconstruction of the dermis before cultured epithelial sheet grafting [6]. However, cultured epithelium grafting results in good results after careful excision of the contracted area, taking care not to cause significant invasion to the deep dermis at the contracted area [17]. In particular, the contracted area after cultured epithelium grafting softens, improving its appearance and texture, and sometimes improving its functionality has also been reported [18]. This softening of the contracted area by cultured epithelium grafting is thought to be due to the metabolism of dermal connective tissue (mainly collagen). Epithelial cells are known to secrete many kinds of proteases in large amounts, and when preparing artificial skin, reconstructed with the dermis component containing cultured epithelial cells, the collagen matrix, fibrin, etc., proteases degrade the dermal structure, making it essential to add a protease inhibitor [19,20].

Treatment of acne scars with a cultured epithelial sheet

The therapy of acne scars using a cultured epithelial sheet grafting performed in this study is not just for the purpose of skin grafting around the excision. Since the grafted epithelium may have been affecting the structure of the skin (especially the dermis), histological examination will be important in the future. The reason is proteases, which are secreted by epithelial cells, degrade excess connective tissue at the contracture site and reduce the surrounding tension, which is expected to soften the scar.

When abrading the scar area, careful excision of the contracted area and FGF-2 was used to promote regeneration of the dermal structure in the deeply damaged area. As a result, the cultured epithelial sheet took well after grafting, and satisfactory results were obtained in terms of cosmetic appearance. This good take of cultured epithelium might also be derived from the regeneration of dermis as well as thin layer abrasion.

However, this case report is limited to short-term results. Long-term observation will be required in the future, including the possibility of recurrence. However, in order to prevent recurrence, daily skin care (such as washing the face to keep it clean) is essential, and a lifestyle that avoids stress is also important.

Technical limitations of this study

These results are case reports, and it is not possible to objectively evaluate whether this technique is superior to the current standard therapy. However, changes in appearance were observed after grafting, and then patients showed a certain degree of satisfaction with the changes in texture and appearance. In addition, the results of the four cases performed were good, and the treatment period, including postoperative management, was rather simpler than standard medical care.

This study has only shown the possibility of therapy with cultured epithelial sheet grafting. When conducting randomized studies based on these results in the future, it will be necessary to set quantifiable criteria.

Even though the number of cases may be limited in the future, it may be possible to evaluate this by conducting an RCT comparing two groups, one standard therapy and one cultured epithelial sheet grafting, or by conducting a meta-analysis comparing this technique with conventional therapy and long-term follow-up.

## Conclusions

Initially, cultured epithelial sheet grafting was a life-saving technique for severe diseases accompanied by extensive skin defects, but by utilizing the characteristics of epithelial cells, it may be possible to apply it not only as a skin substitute but also to the treatment of chronic skin diseases. In particular, it may be useful not only for improving functionality but also for aesthetic intention.
